# Factors of Stress Concentration around Spherical Cavity Embedded in Cylinder Subjected to Internal Pressure

**DOI:** 10.3390/ma14113057

**Published:** 2021-06-03

**Authors:** Mechri Abdelghani, Ghomari Tewfik, Maciej Witek, Djouadi Djahida

**Affiliations:** 1 Mechanical Engineering Faculty, University of Sciences and Technology, Mohamed Boudiaf (USTOMB), BP 1505, El M’naouer, Oran 31000, Algeria; abdelghani.mechri@univ-usto.dz (M.A.); tewfikghomari@yahoo.com (G.T.); djahida.djouadi@univ-usto.dz (D.D.); 2 Composite Structures and Innovative Materials Laboratory (LSCMI), University of Sciences and Technology, Mohamed Boudiaf (USTOMB), BP 1505, El M’naouer, Oran 31000, Algeria; 3 Aeronautics and Propulsive Systems Laboratory (LASP), University of Sciences and Technology, Mohamed Boudiaf (USTOMB), BP 1505, El M’naouer, Oran 31000, Algeria; 4 Gas Engineering Group, Warsaw University of Technology, 20 Nowowiejska St., 00-653 Warsaw, Poland

**Keywords:** pressurized cylinder, spherical cavity, liner-elastic stress analysis, stress concentration factors, analytical solution, numerical simulation results

## Abstract

In this paper, an accurate distribution of stress as well as corresponding factors of stress concentration determination around a spherical cavity, which is considered as embedded in a cylinder exposed to the internal pressure only, is presented. This approach was applied at three main meridians of the porosity by combining the Eshelby’s equivalent inclusion method with Mura and Chang’s methodology employing the jump condition across the interface of the cavity and matrix, respectively. The distribution of stresses around the spherical flaw and their concentration factors were formulated in the form of newly formulated analytical relations involving the geometric ratio of the cylinder, such as external radius and thickness, the angle around the cavity, depth of the porosity, as well as the material Poisson ratio. Subsequently, a comparison of the analytical results and the numerical simulation results is applied to validate obtained results. The results show that the stress concentration factors (SCFs) are not constant for an incorporated flaw and vary with both the porosity depth and the Poisson ratio, regardless of whether the cylinder geometric ratio is thin or thick.

## 1. Introduction

Pressurized structures, such as pipelines and piping systems, are a reliable and inexpensive way to transport energy products in the oil and gas industry [1,2]. Commonly, the cylindrical shells are manufactured according to different specifications depending on the service conditions in which they are supposed to be used. Low alloy steel is the most frequently applied material, owing to the mechanical properties which satisfy the growing demand for high-strength pipes in the oil and gas industry [3,4,5]. Although the usage of pipelines is highly favored, they are sensitive due to several factors that often weaken their ability to withstand the internal pressure. Thus, the load carrying capacity often leads to the rupture of pipelines and systems of tubes, which results in serious environmental, social, and economic consequences for the countries. It is inevitable that the welded joints of metal structures contain imperfections during the construction phase. Therefore, welded cylinders tend to contain typical defects such as cracks, cavities, solid inclusions, and others, see Singh [6]. Therefore, to ensure safer operation of the cylindrical structures, the study of flaws has become an important factor for an adequate assessment of the latter. Unfortunately, volumetric defects, such as cavities and solid inclusions, are generally either rejected or accepted as a result of non-destructive testing based on the acceptability criteria of relevant codes, for example, British Standard BS 7910:2015 [7] and American Petroleum Institute standard 579, see Anderson and Osage [8]. As a result, these codes require appropriate evaluation methods for assessing volumetric defects. Commonly, the defects embedded in the materials are called inclusions and are classified by Mura, R. & Ting, T.C.T [9] as homogeneous inclusions, inhomogeneous inclusions, and inhomogeneities. Thus, a homogeneous inclusion demonstrates the same mechanical characteristics as the matrix; however, it is characterized by an eigenstrain referring to thermal expansion or the material transformation phase. On the other hand, inhomogeneous inclusions present different mechanical properties and eigenstrain than the matrix. Inhomogeneity zone demonstrates also various mechanical properties compared to the matrix, however, is characterized by an eigenstrain.

Since the 1930s, many theoretical studies have been conducted to elucidate the effect of the presence of one or more inclusions in an infinite isotropic-elastic medium subjected to an external stress field. Among the important works published earlier, the works by Eshelby, J.D. [10,11] are cited in the present paper. Eshelby, J.D. developed a method called the Equivalent Inclusion Method (EIM) in which inhomogeneity is reduced to a homogeneous inclusion leading to the resolution of the stress field inside and outside an ellipsoidal inclusion embedded in an infinite isotropic-elastic medium. Since its development, EIM has been used to determine the stress field in innumerable studies on inclusions of different shapes and properties. More details related to EIM are discussed by Christensen [12], Mura and Ting [9], Murakami [13], Nemat-Nasser and al. [14], Qu &Cherkaoui [15], and Li & Gao [16]. However, the assessment of defect micromechanics of a single inclusion has been extended to the interaction of two or more inclusions in an infinite medium, see Moschovidis& Mura [17], Fond, C and al. [18], Sabina, F.J and al. [19], Benedikt, and Lewis and Rangaswamy [20].

**Figure 1:**
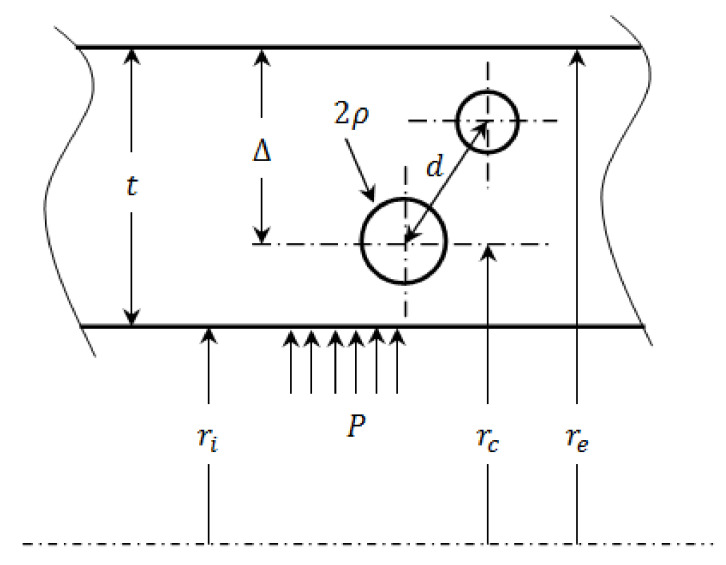
Distance between two spherical inclusions of different radii embedded in an isotropic-elastic material.

As indicated in Figure 1, the above studies have shown that the interaction between two adjacent inclusions of various radii becomes negligible if the inter-inclusions distance *d* exceeds the largest inclusion radius at least fivefold.

Studies of inhomogeneous materials for a certain number of technological applications are focused on the behavior of inclusions when they are close to a half-space. The effect of a half-space on an inclusion is also clarified in numerous studies, among others, Ru [21], Sun and Peng [22], He and Li [23], Seo and Mura [24], and Mi and Kouris [25]. In these studies, the disturbance of the stress field around an inclusion is negligible at a distance determined by porosity depth Δ exceeding the radius of the inclusion fivefold Δ≥5ρ. Thus, an inclusion can be considered as isolated in a semi-infinite matrix if its location relative to the nearest inclusion or a half-plane is greater than 5ρ (Figure 1). The objective of the present study is to determine stress concentration factors (SCFs) around a spherical cavity embedded in a cylinder subjected to the internal pressure. Therefore, such a porosity is subjected to a triaxial stress field associated with the applied internal pressure. In order to resolve the stress discontinuity around the flaw/matrix interface, EIM method is used with the Mura and Cheng jump condition [26] using a linear-elastic constitutive law for an isotropic homogeneous material, assuming that the porosity is an inhomogeneity with negligible mechanical characteristics. In order to satisfy the matrix infinity condition, the location of the flaw is assumed to be at least 5ρ from the nearest internal or external surface of the cylinder. These hypotheses lead to the determination of the stress distribution and the stress concentration factors $K_{t}$ around the three main meridians of the spherical cavity. Subsequently, a finite element analysis (FEA) using ANSYS is carried out to validate the proposed approach. The contribution of the present paper is a useful analytical solution to evaluate an embedded welding defect such as cavity or porosity. This is the first time that Eshelby’s theory has been used to describe the stress field around inclusions and porosities in welded joints in a cylinder under internal pressure and it can be treated as novelty of the current paper.

## 2. Theoretical Background

### 2.1. Eshelby’s Homogenization Principle

**Figure 2:**
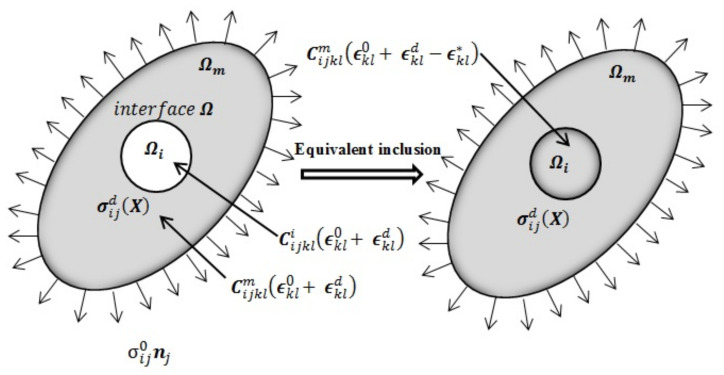
Eshelby’s equivalent inclusion method.

Eshelby’s problem, as shown in Figure 2, is a case in which an elastic inclusion $\Omega_{i}$ with an elastic stiffness tensor $C_{ijkl}^{i}$ is embedded inside an infinite and elastic matrix $\Omega_{m}$ having a different elastic stiffness tensor $C_{ijkl}^{m}$. Then, the overall solid body (inclusion and matrix) is subjected to a uniform deformation $\epsilon_{ij}^{0}$ related to the applied stress field $\sigma_{ij}^{0}$. To resolve this issue, Eshelby introduced the concept of equivalent inclusion by replacing the heterogeneous inclusion with another homogeneous inclusion of the same form with the addition of an equivalent eigenstrain $\epsilon_{ij}^{*}$ to achieve the same stress field and deformation as in the case of the heterogeneous inclusion (Figure 2). The added eigenstrain $\epsilon_{ij}^{*}$ can be expressed as follows:

(1) $$\epsilon_{ij}^{*}\left(X\right)=\left\{\begin{array}{c} 0\;\;\;,\;\;\;\forall \;X\in \Omega_{m}\\ \epsilon_{ij}^{*}\;\;\;,\;\forall \;X\in \Omega_{i} \end{array}\right.$$

In the case illustrated in Figure 2, the difference in the elastic stiffness tensor between the matrix and the inclusion disrupts the total stress and strain fields by the amounts of $\sigma_{ij}^{d}\left(X\right)$ and $\epsilon_{ij}^{d}\left(X\right)$. Taking into consideration the above statement, the distribution of the total stress and strain in solid V is described by the following equations:

(2) $$\sigma_{ij}\left(X\right)=\sigma_{ij}^{0}+\sigma_{ij}^{d}\left(X\right)$$

(3) $$\epsilon_{ij}\left(X\right)=\epsilon_{ij}^{0}+\epsilon_{ij}^{d}\left(X\right)$$

The stress $\sigma_{ij}\left(X\right)$ can be correlated with the stiffness tensor of the matrix $C_{ijkl}^{m}$ and the inclusion $C_{ijkl}^{i}$ using the following relations:

(4) $$\sigma_{ij}\left(X\right)=\left\{\begin{array}{c} C_{ijkl}^{m}\left(\epsilon_{kl}^{0}+\epsilon_{kl}^{d}\left(X\right)\right),\;\;X\in \Omega_{m}\\ C_{ijkl}^{i}\left(\epsilon_{kl}^{0}+\epsilon_{kl}^{d}\left(X\right)\right),\;\;X\in \;\Omega_{i} \end{array}\right.$$

In Equation (4), the stiffness moduli $C_{ijkl}^{m}$ and $C_{ijkl}^{i}$ are given by the following expression:

(5) $$C_{ijkl}=\lambda \delta_{ij}\delta_{kl}+\mu \left(\delta_{ik}\delta_{jl}+\delta_{il}\delta_{jk}\right)$$

where the first and the second Lame’s coefficients, λ and μ, are connected to the isotropic-elastic modulus E and Poisson’s ratio ν, respectively, using the expressions

(6) $$\lambda =\frac{E\;\nu }{\left(1+\nu \right)\left(1-2\nu \right)}$$

(7) $$\mu =\frac{E\;}{2\left(1+\nu \right)}$$

By applying the Eshelby’s equivalent inclusion principle, it is possible to express the total stress field as

(8) $$\sigma_{ij}\left(X\right)=\left\{\begin{array}{c} C_{ijkl}^{m}\left(\epsilon_{kl}^{0}+\epsilon_{kl}^{d}\left(X\right)\right),\;\;\forall \;X\in \Omega_{m}\\ C_{ijkl}^{m}\left(\epsilon_{kl}^{0}+\epsilon_{kl}^{d}\left(X\right)-\epsilon_{kl}^{*}\left(X\right)\right),\;\;\forall \;X\in \;\Omega_{i} \end{array}\right.$$

The Eshelby’s tensor, $S_{ijkl}$,which was defined in original studies by Eshelby, J.D. [10,11] and, in more detail, in Mura [9], connects deformation $\epsilon_{ij}^{d}$ in the inclusionwith the eigenstrain $\epsilon_{ij}^{*}$ through $\epsilon_{ij}^{d}\left(X\right)=S_{ijkl}\epsilon_{kl}^{*}$, $\forall \;X\in \Omega_{i}$; thus, the expression of the total stress field is

(9) $$\sigma_{ij}\left(X\right)=\left\{\begin{array}{c} C_{ijkl}^{m}\left(\epsilon_{kl}^{0}+\epsilon_{kl}^{d}\left(X\right)\right),\;\;\forall \;X\in \Omega_{m}\\ C_{ijkl}^{m}\left(\epsilon_{kl}^{0}+S_{klmn}\epsilon_{mn}^{*}\left(X\right)-\epsilon_{kl}^{*}\left(X\right)\right),\;\;\forall \;X\in \;\Omega_{i} \end{array}\right.$$

Comparison of Equations (4) and (9) leads to the following fundamental relationship incorporating inhomogeneity into the matrix:

(10) $$C_{ijkl}^{i}\left(\epsilon_{kl}^{0}+S_{klmn}\epsilon_{mn}^{*}\right)=C_{ijkl}^{m}\left(\epsilon_{kl}^{0}+S_{klmn}\epsilon_{mn}^{*}-\epsilon_{kl}^{*}\right)$$

From Formula (10), the eigenstrain field $\epsilon_{ij}^{*}$ in $\Omega_{i}$ can be solved if the stress field $\sigma_{ij}^{0}$ and deformation $\epsilon_{ij}^{0}$ are given.

### 2.2. Jump Condition across Interface Ω

It is worth noting that the stress field presents a discontinuity across the boundary of the inclusion. Having the eigenstrain $\epsilon_{ij}^{*}$ known, Mura and Cheng [26] solved stress discontinuity across the interface Ω, by deducing the stress jump $\Delta \sigma_{ij}\left(X\right)$ through the interface Ω, with the following expression:

(11) $$\Delta \sigma_{ij}\left(X\right)=\sigma_{ij}^{out}-\sigma_{ij}^{in}=C_{ijkl}^{m}\left[-C_{pqmn}^{m}\epsilon_{mn}^{*}\left(X\right)M_{kp}n_{q}n_{l}+\epsilon_{kl}^{*}\left(X\right)\right],\;\;\;\;\;\forall \;X\in \Omega \;$$

where

(12) $$M_{kp}=\frac{1}{\mu_{m}}\left[\delta_{kp}-\frac{n_{k}n_{p}}{2\left(1-\nu_{m}\right)}\right]$$

**Figure 3:**
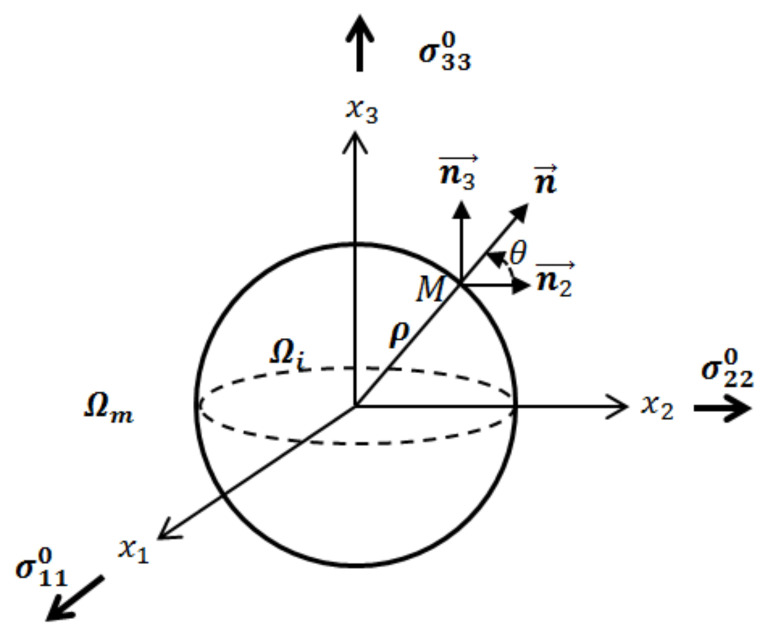
Vector position of point M in plane $x_{1}=0$ ($n_{1}=0$).

In Equation (12), $n_{k}$ and $n_{p}$ are the projections of the unit vector n on $x_{k}$ and $x_{p}$ axes, as indicated in Figure 3. $\delta_{ij}$ is Kronecker’s delta operator, $\mu_{m}$ and $\nu_{m}$ the shear modulus and Poisson’s ratio of the matrix $\Omega_{m}$, respectively.

For an eigenstrain field, $\epsilon_{ij}^{*},$ given in an embedded inclusion in an infinite isotropic medium, and subjected to a field stress $\sigma_{ij}^{0}$, Equation (11) can be used to solve the stress field $\sigma_{ij}\left(X\right)=\sigma_{ij}^{out}$, ∀ X∈Ω around the inclusion:

(13) $$\sigma_{ij}\left(X\right)=\sigma_{ij}^{in}+C_{ijkl}^{m}\left[-C_{pqmn}^{m}\epsilon_{mn}^{*}\left(X\right)M_{kp}n_{q}n_{l}+\epsilon_{kl}^{*}\left(X\right)\right],\;\;\;\;\;\forall \;X\in \Omega$$

### 2.3. Inhomogeneity in the Form of Spherical Cavity

If inhomogeneity $\Omega_{i}$ is regarded as a spherical porosity of radius ρ, its characteristics $\nu_{i}$, ${µ}_{i}$, and $\lambda_{i}$ can be neglected and, according to Formula (5), the stiffness tensor of the cavity is $C_{ijkl}^{i}=0$. Thus, $\sigma_{ij}^{in}=0$ and then Equations (10) and (11) can be reduced to

(14) $$\epsilon_{kl}^{0}+(S_{klmn}-I_{klmn})\epsilon_{mn}^{*}=0$$

(15) $$\sigma_{ij}=C_{ijkl}^{m}\left[-C_{pqmn}^{m}\epsilon_{mn}^{*}\left(X\right)M_{kp}n_{q}n_{l}+\epsilon_{kl}^{*}\left(X\right)\right];\;\;\;\;\;\;\;\forall \;X\in \Omega$$

where the fourth-ranked unit tensor $I_{klmn}=\left(\delta_{km}\delta_{ln}+\delta_{kn}\delta_{lm}\right)/3$.

## 3. Spherical Cavity Embedded in a Cylinder Wall

**Figure 4:**
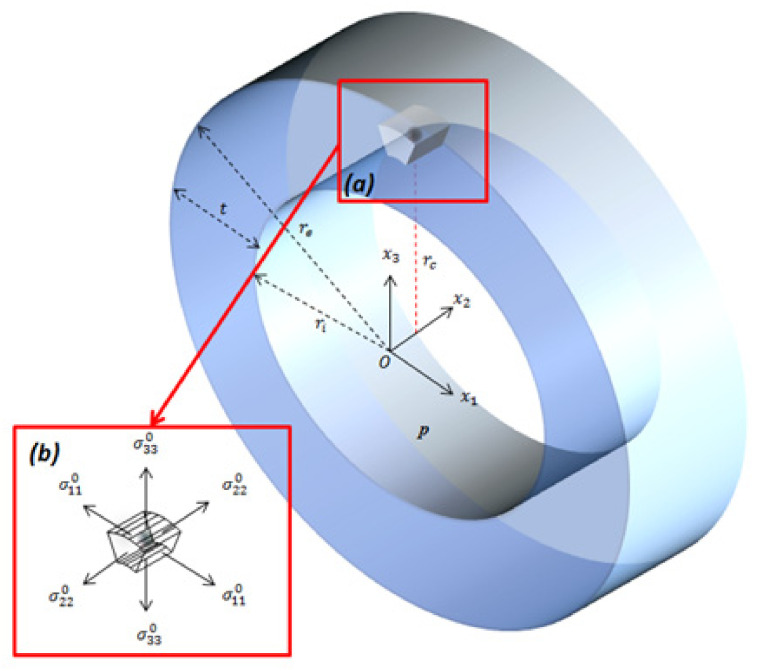
Spherical cavity embedded in the weld of an isotropic-elastic long cylinder: (**a**) elementary solid volume surrounding the porosity and (**b**) components of the average stress acting on the faces of the elementary volume of the solid body.

The long tube shown in Figure 4 is considered to be isotropic-elastic, and its inner and outer radii are $r_{i}$ and $r_{e}$, respectively. A cavity with radius ρ can be considered as incorporated if its location $r_{c}$ from the cylinder axis satisfies the following condition:

(16) $$r_{i}+5\rho \leq r_{c}\leq r_{e}-5\rho$$

When the cylindrical shell is subjected to the internal pressure P, the average components of the nominal stress in the circumferential, axial and radial directions, acting on the surfaces of the elementary volume containing the cavity (Figure 4b), can be expressed using the below Lame equations, see Timoshenko and Goodier [27]:

(17) $$\sigma_{11}^{0}=P\frac{r_{i}^{2}}{r_{e}^{2}-r_{i}^{2}}\left(1+\frac{r_{e}^{2}}{r_{c}^{2}}\right)$$

(18) $$\sigma_{22}^{0}=P\frac{r_{i}^{2}}{r_{e}^{2}-r_{i}^{2}}$$

(19) $$\sigma_{33}^{0}=P\frac{r_{i}^{2}}{r_{e}^{2}-r_{i}^{2}}\left(1-\frac{r_{e}^{2}}{r_{c}^{2}}\right)$$

The corresponding average components of deformation in the circumferential, axial and radial directions are obtained by substitution of Equations (17)–(19) into the formula of the strain tensor $\epsilon_{ij}^{0}=\left(1+\nu_{m}/E_{m}\right)\sigma_{ij}^{0}-\left(\nu_{m}/E_{m}\right)\sigma_{kk}^{0}\delta_{ij}$, as follows:

(20) $$\epsilon_{11}^{0}=\frac{P}{E_{m}}\frac{r_{i}^{2}}{\left(r_{e}^{2}-r_{i}^{2}\right)}\left[\left(1+\nu_{m}\right)\frac{r_{e}^{2}}{r_{c}^{2}}+\left(1-2\nu_{m}\right)\right]$$

(21) $$\epsilon_{22}^{0}=\frac{P}{E_{m}}\frac{r_{i}^{2}}{\left(r_{e}^{2}-r_{i}^{2}\right)}\left(1-2\nu_{m}\right)$$

(22) $$\epsilon_{33}^{0}=-\frac{P}{E_{m}}\frac{r_{i}^{2}}{\left(r_{e}^{2}-r_{i}^{2}\right)}\left[\left(1+\nu_{m}\right)\frac{r_{e}^{2}}{r_{c}^{2}}-\left(1-2\nu_{m}\right)\right]$$

By applying to Formula (14) the tensor’s summation rule, the explicit expressions of eigenstrain components $\epsilon_{ij}^{*}$ are obtained in the inclusion and the equators $x_{1}=0$, $x_{2}=0$ and $x_{3}=0$:

(23) $$\epsilon_{11}^{*}=\left[C_{1}\epsilon_{11}^{0}-C_{2}\epsilon_{22}^{0}+C_{2}\epsilon_{33}^{0}\right]/C_{0}$$

(24) $$\epsilon_{22}^{*}=\left[2C_{3}\epsilon_{11}^{0}+\left(C_{4}+C_{5}C_{0}\right)\epsilon_{22}^{0}+\left(C_{4}-C_{5}C_{0}\right)\epsilon_{33}^{0}\right]/2C_{0}$$

(25) $$\epsilon_{33}^{*}=\left[2C_{3}\epsilon_{11}^{0}+\left(C_{4}-C_{5}C_{0}\right)\epsilon_{22}^{0}+\left(C_{4}+C_{5}C_{0}\right)\epsilon_{33}^{0}\right]/2C_{0}$$

where the dimensionless constants $C_{0}$−$C_{5}$ in Equations from (23) to (25) are defined by

$$C_{0}=-1+\left(S_{1111}+S_{2222}+S_{2233}\right)-\left[S_{1111}\left(S_{2222}+S_{2233}\right)-2S_{1122}S_{2211}\right]\;C_{1}=S_{2222}+S_{2233}-1\;C_{2}=S_{1122}C_{3}=-S_{1122}\;C_{4}=S_{1111}\;C_{5}=1/\left(S_{2233}-S_{2222}\right)$$

where the components of the Eshelby’s tensor $S_{ijkl}$ for the spherical inclusion (as shown in Appendix A.1) are derived from the following expression:

(26) $$S_{ijkl}=-\frac{\left(1-5\nu_{m}\right)}{15\left(1-\nu_{m}\right)}\delta_{ij}\delta_{kl}+\frac{\left(4-5\nu_{m}\right)}{15\left(1-\nu_{m}\right)}\left(\delta_{ik}\delta_{jl}+\delta_{il}\delta_{jk}\right)$$

In order to express the eigenstrain in the spherical cavity in terms of the applied pressure P, the mechanical characteristics and dimensions of the cylinder, Equations (20)–(22) are replaced in Equations (23)–(25) to provide

(27) $$\epsilon_{11}^{*}=\frac{3}{2}\frac{P}{E_{m}}\frac{r_{i}^{2}}{\left(r_{e}^{2}-r_{i}^{2}\right)}\left(1-\nu_{m}\right)\left[10\frac{r_{e}^{2}}{r_{c}^{2}}\left(\frac{1+\nu_{m}}{7-5\nu_{m}}\right)+1\right]$$

(28) $$\epsilon_{22}^{*}=\frac{3}{2}\frac{P}{E_{m}}\frac{r_{i}^{2}}{\left(r_{e}^{2}-r_{i}^{2}\right)}\left(1-\nu_{m}\right)$$

(29) $$\epsilon_{33}^{*}=-\frac{3}{2}\frac{P}{E_{m}}\frac{r_{i}^{2}}{\left(r_{e}^{2}-r_{i}^{2}\right)}\left(1-\nu_{m}\right)\left[10\frac{r_{e}^{2}}{r_{c}^{2}}\left(\frac{1+\nu_{m}}{7-5\nu_{m}}\right)-1\right]$$

As indicated in Appendix A.2, the successive substitution of the eigenstrain expression $\epsilon_{ij}^{*}$ given by Equations (27)–(29) in Formula (15) leads to the determination of the stress components around the cavity in the equators $x_{1}=0$, $x_{2}=0\;$ and $x_{3}=0$. The solution is provided in terms of P, angle θ, $\nu_{m}$ and dimensions of the cylinder, as follows:

- stress components in equator $x_{1}=0$:

(30) $$\sigma_{11}=\frac{3}{2}\;P\frac{r_{i}^{2}}{\left(r_{e}^{2}-r_{i}^{2}\right)}\left[10\;\frac{r_{e}^{2}}{r_{c}^{2}}\frac{\left(\nu_{m}\cos^{2}\theta -1\right)}{\left(-7+5\nu_{m}\right)}+1\right]$$

(31) $$\sigma_{22}=\frac{3}{2}P\frac{r_{i}^{2}}{\left(r_{e}^{2}-r_{i}^{2}\right)}\sin^{2}\theta \left[10\;\frac{r_{e}^{2}}{r_{c}^{2}}\frac{\left(\cos^{2}\theta -\nu_{m}\right)}{\left(-7+5\nu_{m}\right)}+1\right]$$

(32) $$\sigma_{33}=\frac{3}{2}P\frac{r_{i}^{2}}{\left(r_{e}^{2}-r_{i}^{2}\right)}\cos^{2}\theta \left[10\;\frac{r_{e}^{2}}{r_{c}^{2}}\frac{\left(\cos^{2}\theta -\nu_{m}\right)}{\left(-7+5\nu_{m}\right)}+1\right]$$

- stress components in equator $x_{2}=0$:

(33) $$\sigma_{11}=\frac{3}{2}P\frac{r_{i}^{2}}{\left(r_{e}^{2}-r_{i}^{2}\right)}\sin^{2}\theta \left[10\;\frac{r_{e}^{2}}{r_{c}^{2}}\frac{\left(2\cos^{2}\theta -1\right)}{\left(-7+5\nu_{m}\right)}+1\right]$$

(34) $$\sigma_{22}=\frac{3}{2}\;P\frac{r_{i}^{2}}{\left(r_{e}^{2}-r_{i}^{2}\right)}\left[10\;\frac{r_{e}^{2}}{r_{c}^{2}}\nu_{m}\frac{\left(2\cos^{2}\theta -1\right)}{\left(-7+5\nu_{m}\right)}+1\right]$$

(35) $$\sigma_{33}=\frac{3}{2}P\frac{r_{i}^{2}}{\left(r_{e}^{2}-r_{i}^{2}\right)}\cos^{2}\theta \left[10\;\frac{r_{e}^{2}}{r_{c}^{2}}\frac{\left(2\cos^{2}\theta -1\right)}{\left(-7+5\nu_{m}\right)}+1\right]$$

- stress components in equator $x_{3}=0$:

(36) $$\sigma_{11}=\frac{3}{2}P\frac{r_{i}^{2}}{\left(r_{e}^{2}-r_{i}^{2}\right)}\sin^{2}\theta \left[10\;\frac{r_{e}^{2}}{r_{c}^{2}}\frac{\left(\sin^{2}\theta -\nu_{m}\right)}{\left(7-5\nu_{m}\right)}+1\right]$$

(37) $$\sigma_{22}=\frac{3}{2}P\frac{r_{i}^{2}}{\left(r_{e}^{2}-r_{i}^{2}\right)}\cos^{2}\theta \left[10\;\frac{r_{e}^{2}}{r_{c}^{2}}\frac{\left(\sin^{2}\theta -\nu_{m}\right)}{\left(7-5\nu_{m}\right)}+1\right]$$

(38) $$\sigma_{33}=\frac{3}{2}P\frac{r_{i}^{2}}{\left(r_{e}^{2}-r_{i}^{2}\right)}\left[10\;\frac{r_{e}^{2}}{r_{c}^{2}}\frac{\left(\nu_{m}\sin^{2}\theta -1\right)}{\left(7-5\nu_{m}\right)}+1\right]$$

## 4. Factors of Stress Concentration around a Cavity within the Tube Wall

In order to deduce an explicit form for $K_{t}$ factors, position $r_{c}$ can be decomposed into two parts $r_{c}=r_{e}-\Delta$, where Δ is the depth of the porosity within the cylinder wall. Thus, if the geometric ratio is considered as $\kappa =r_{e}/t,\;\;r_{e}/r_{c}$ ratio can be expressed as follows:

(39) $$\frac{r_{e}}{r_{c}}=\frac{\kappa }{\kappa -\Delta /t}$$

By substituting Formula (39) into Equations (30)–(38), the stress components $\sigma_{11}$, $\sigma_{22}$, and $\sigma_{33}$ within three meridians can be fully described in terms of $\nu_{m}$, the angle θ, the dimensionless depth of the cavity $\left(\Delta /t\right)$, and the geometric ratio κ of the cylinder. Therefore,

- stress components in equator $x_{1}=0$:

(40) $$\sigma_{11}=\frac{3}{2}\frac{\sigma_{11}^{0}}{\left[1+{\left(\frac{\kappa }{\kappa -\left(\Delta /t\right)}\right)}^{2}\right]}\left[10\;{\left(\frac{\kappa }{\kappa -\left(\Delta /t\right)}\right)}^{2}\frac{\left(\nu_{m}\cos^{2}\theta -1\right)}{\left(-7+5\nu_{m}\right)}+1\right]$$

(41) $$\sigma_{22}=\frac{3}{2}\sigma_{22}^{0}\;sin^{2}\theta \left[10\;{\left(\frac{\kappa }{\kappa -\left(\Delta /t\right)}\right)}^{2}\frac{\left(\cos^{2}\theta -\nu_{m}\right)}{\left(-7+5\nu_{m}\right)}+1\right]$$

(42) $$\sigma_{33}=\frac{3}{2}\frac{\sigma_{33}^{0}}{\left[1-{\left(\frac{\kappa }{\kappa -\left(\Delta /t\right)}\right)}^{2}\right]}\cos^{2}\theta \left[10\;{\left(\frac{\kappa }{\kappa -\left(\Delta /t\right)}\right)}^{2}\frac{\left(\cos^{2}\theta -\nu_{m}\right)}{\left(-7+5\nu_{m}\right)}+1\right]$$

- stress components in equator $x_{2}=0$:

(43) $$\sigma_{11}=\frac{3}{2}\frac{\sigma_{11}^{0}}{\left[1+{\left(\frac{\kappa }{\kappa -\left(\Delta /t\right)}\right)}^{2}\right]}\sin^{2}\theta \left[10\;{\left(\frac{\kappa }{\kappa -\left(\Delta /t\right)}\right)}^{2}\frac{\left(2\cos^{2}\theta -1\right)}{\left(-7+5\nu_{m}\right)}+1\right]$$

(44) $$\sigma_{22}=\frac{3}{2}\sigma_{22}^{0}\left[10\;{\left(\frac{\kappa }{\kappa -\left(\Delta /t\right)}\right)}^{2}\nu_{m}\frac{\left(2\cos^{2}\theta -1\right)}{\left(-7+5\nu_{m}\right)}+1\right]$$

(45) $$\sigma_{33}=\frac{3}{2}\frac{\sigma_{33}^{0}}{\left[1-{\left(\frac{\kappa }{\kappa -\left(\Delta /t\right)}\right)}^{2}\right]}\cos^{2}\theta \left[10\;{\left(\frac{\kappa }{\kappa -\left(\Delta /t\right)}\right)}^{2}\frac{\left(2\cos^{2}\theta -1\right)}{\left(-7+5\nu_{m}\right)}+1\right]$$

- stress components in equator $x_{3}=0$:

(46) $$\sigma_{11}=\frac{3}{2}\frac{\sigma_{11}^{0}}{\left[1+{\left(\frac{\kappa }{\kappa -\left(\Delta /t\right)}\right)}^{2}\right]}\sin^{2}\theta \left[10\;{\left(\frac{\kappa }{\kappa -\left(\Delta /t\right)}\right)}^{2}\frac{\left(\sin^{2}\theta -\nu_{m}\right)}{\left(7-5\nu_{m}\right)}+1\right]$$

(47) $$\sigma_{22}=\frac{3}{2}\;\sigma_{22}^{0}\cos^{2}\theta \left[10\;{\left(\frac{\kappa }{\kappa -\left(\Delta /t\right)}\right)}^{2}\frac{\left(\sin^{2}\theta -\nu_{m}\right)}{\left(7-5\nu_{m}\right)}+1\right]$$

(48) $$\sigma_{33}=\frac{3}{2}\frac{\sigma_{33}^{0}}{\left[1-{\left(\frac{\kappa }{\kappa -\left(\Delta /t\right)}\right)}^{2}\right]}\left[10\;{\left(\frac{\kappa }{\kappa -\left(\Delta /t\right)}\right)}^{2}\frac{\left(\nu_{m}\sin^{2}\theta -1\right)}{\left(7-5\nu_{m}\right)}+1\right]$$

## 5. Numerical Validation of the Presented Methodology

**Figure 5:**
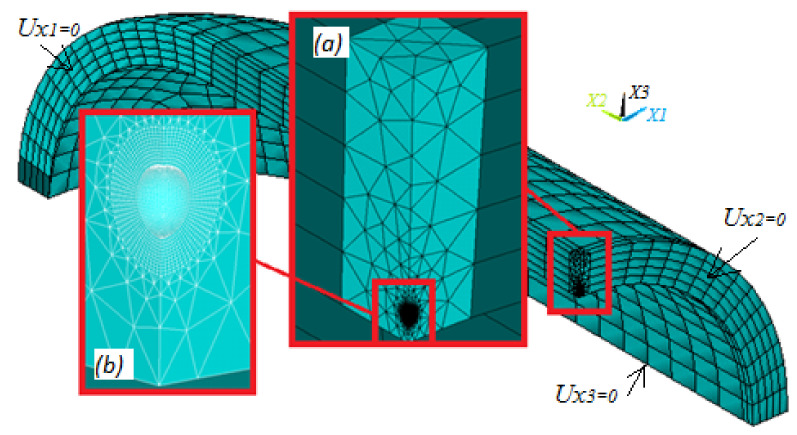
A typical mesh in the pipe containing a spherical cavity: (**a**) depth of the flaw on the thickness of the cylinder and (**b**) magnification of the area occupied by the spherical porosity.

In order to validate the determination of stress components around the three main equators of a spherical cavity identified by Equations (40)–(48), linear-elastic stress analyses were performed using the ANSYS code. The proposed solutions are, thus, validated by means of a comparison with the results obtained by FEA. Figure 5 illustrates the geometry of the solid body used in the numerical analysis, in which the symmetry with respect to x, y, and z planes allowed reducing the numerical model to an eighth model. A fine and mapped mesh (using solid-186 elements) is applied around the porosity, and a relatively coarse mesh for the remaining parts of the cylinder mesh is used as a transition to connect both zones. The following boundary conditions are applied to the eighth model: the movements are constrained in the *x* = 0, *y* = 0, and *z* = 0 planes. However, the model may only expand in the radial direction. The pressure of 14 MPa is applied to the entire inner surface of the numerical model. The material of the pipeline is carbon steel, whose elastic modulus $E_{m}=225\;\mathrm{GPa}$ and Poisson module $\nu_{m}=0.26$. The radii of the tube are $r_{e}$ = 155 mm and $r_{i}$ = 150 mm. The focus of this validation was specifically set on the determination of stresses around the main equators of a spherical cavity of radius ρ=0.5 mm. The location of the flaw is selected as a dimensionless depth Δ/t = 0.75 which is related to the highest multi-axial stress condition.

**Figure 6:**
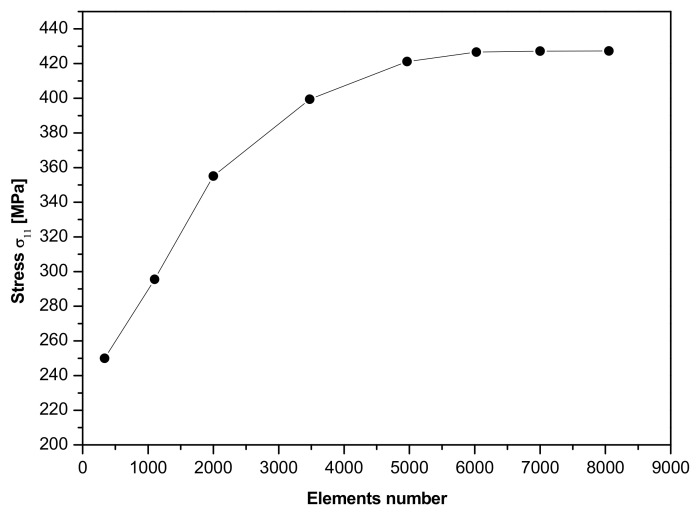
Convergence curve of principal stress $\sigma_{11}$ at angle θ=π/2.

In order to achieve the results that are reliable when using the finite element method, a stress convergence study is carried out. The presented model is required to produce accurate stresses only at defect region; the role of all elements away from the defect region is only to represent the geometry and transmit the applied load. Therefore, local refinement at the defect region is studied and the stresses of interest do not affect the stresses elsewhere. Figure 6 shows the results of the mesh convergence study. Note that the stress asymptotes about a mesh density of around 6500 elements. In the present paper, a mesh density of 8000 elements is used, therefore it would be an appropriate element density to demonstrate the proposed numerical model.

**Figure 7:**
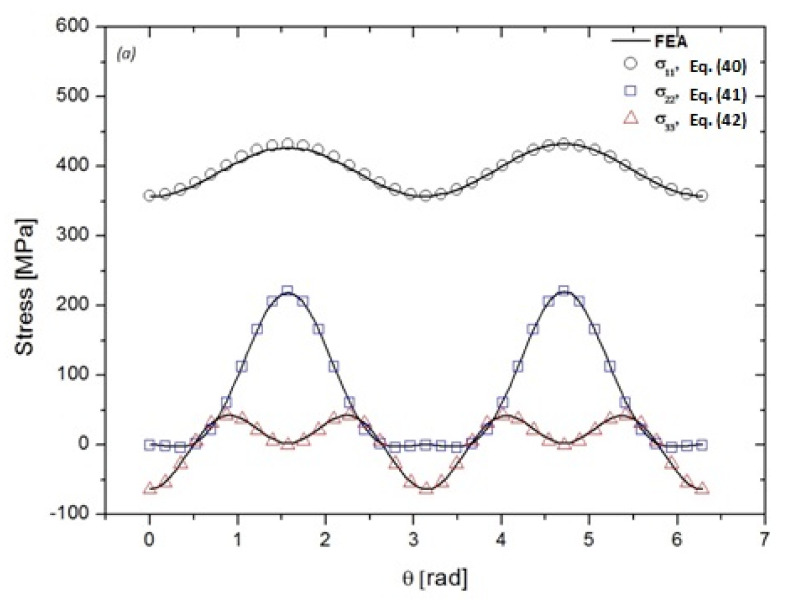

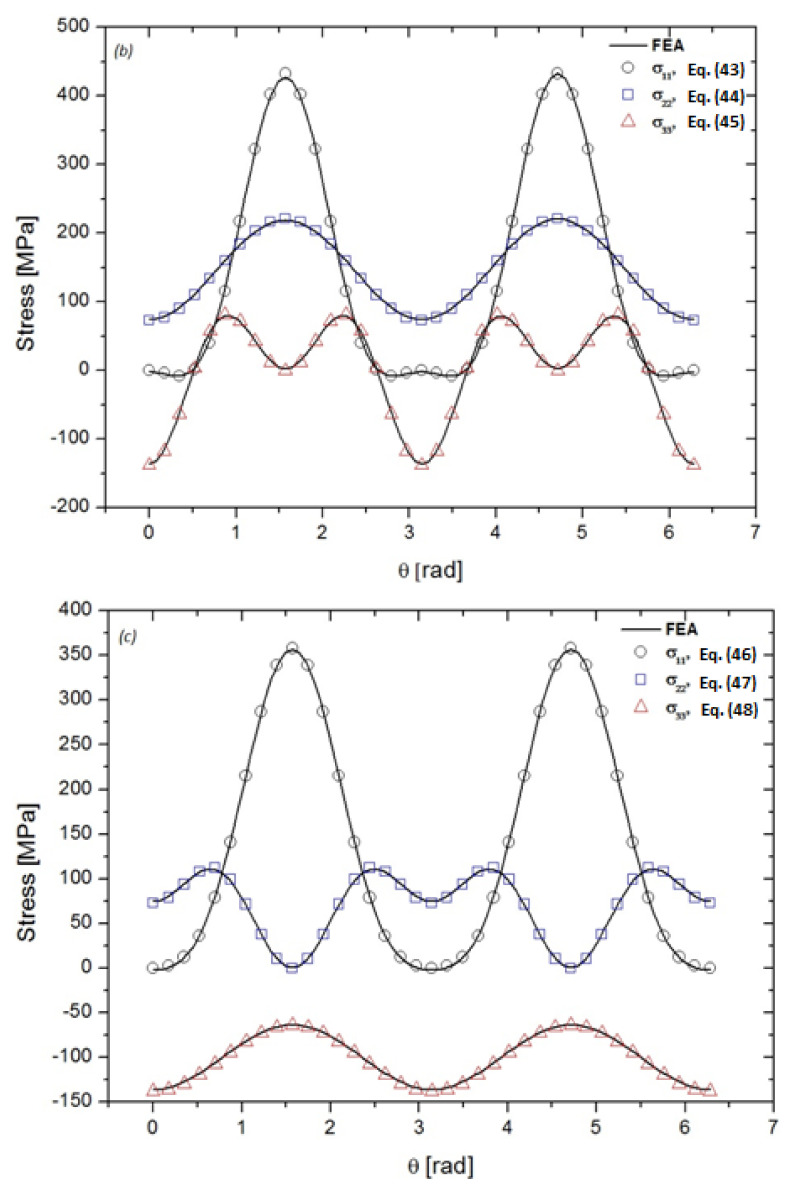
Variations of stress components around the spherical cavity: (**a**) equator $x_{1}=0$, (**b**) equator $x_{2}=0$, and (**c**) equator $x_{3}=0$. Porosity radius ρ=0.5 mm, dimensionless geometric ratio of the cylinder κ=15.5, and dimensionless depth Δ/t=0.75.

**Figure 8:**
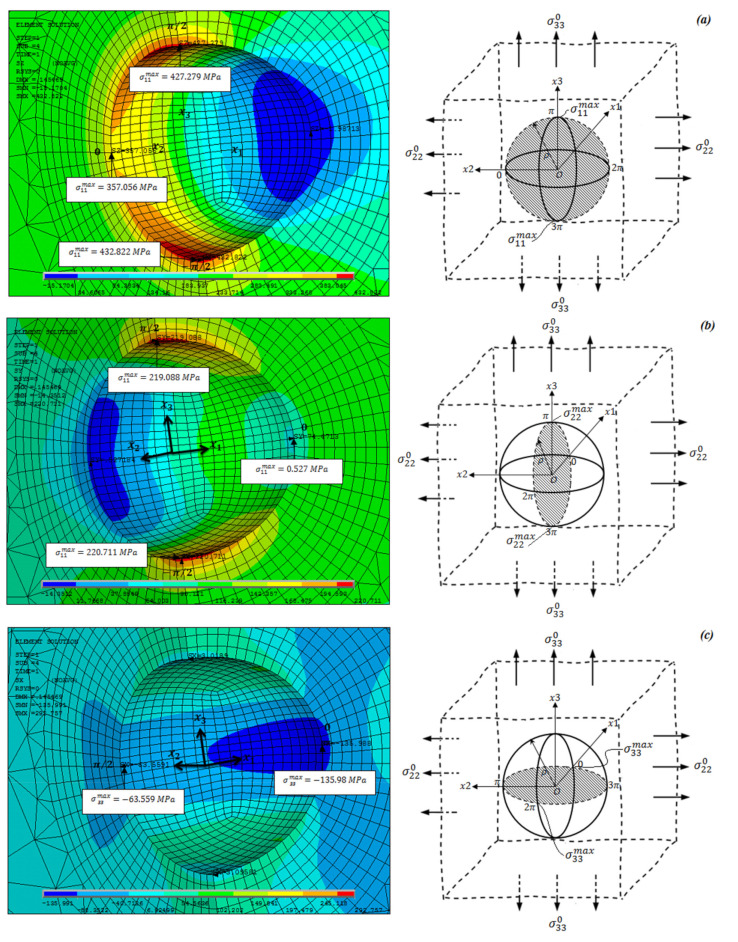
Repartition of the components of stress around the spherical cavity and the stress values at θ=0, π/2, and −π/2, (**a**) component of hoop stress $\sigma_{11}$, (**b**) component of axial stress $\sigma_{22}$, and (**c**) component of radial stress $\sigma_{33}$.

Figure 7a–c compares the evolution of stress components $\sigma_{11}$, $\sigma_{22}$, and $\sigma_{33}$, respectively, obtained with FEM, in equators $x_{1}=0$, $x_{2}=0$, and $x_{3}=0$ with those obtained with Equations (40)–(48). The obtained results correspond to the internal pressure load of 14 MPa. Figure 8a–c shows the stress distribution and locations of the maximum values obtained. As it is shown below, the components of circumferential and axial stresses reached their maximum values at angles θ = π/2 and −π/2, while the axial stress component reaches its maximum value at angles θ = 0 and π. However, a comparison of the analytical estimates and the numerical values indicates a satisfactory agreement with a maximum difference of +1.182%. Therefore, this comparison validates the determination of the stresses around the cavity incorporated in the cylinder.

As indicated above, π/2 and −π/2 are angles at which circumferential and radial stress are concentrated and reach the maximum values. However, Equations (40) and (44) can be reduced to provide expressions of K*_t_*_1_ and K*_t_*_2_, respectively, in the following form:

(49) $$K_{t1}=\frac{3}{2}\frac{1}{\left[1+{\left(\frac{\kappa }{\kappa -\left(\Delta /t\right)}\right)}^{2}\right]}\left[\frac{10}{\left(7-5\nu_{m}\right)}{\left(\frac{\kappa }{\kappa -\left(\Delta /t\right)}\right)}^{2}+1\right]$$

(50) $$K_{t2}=\frac{3}{2}\left[\frac{10}{\left(7-5\nu_{m}\right)}{\left(\frac{\kappa }{\kappa -\left(\Delta /t\right)}\right)}^{2}\nu_{m}+1\right]$$

Similarly, the axial compressive stress reaches its maximum value at angles 0 and π. Thus, K*_t3_* stress concentration factor can be derived from Formula (48) to obtain

(51) $$K_{t3}=\frac{3}{2}\frac{1}{\left[1-{\left(\frac{\kappa }{\kappa -\left(\Delta /t\right)}\right)}^{2}\right]}\left[\frac{10}{\left(5\nu_{m}-7\right)}{\left(\frac{\kappa }{\kappa -\left(\Delta /t\right)}\right)}^{2}+1\right]$$

Table 1 and Table 2 compare the results obtained from Equations (49)–(51) with the results of FEA for three materials with Poisson ratios ν_m_ = 0.25, 0.3, and 0.35. Two geometric ratios for κ have been taken into account: κ = 15.5 for a thin-walled cylinder and κ = 3.1 for a thick tube. For each geometric ratio κ, three various dimensionless porosity depths were examined Δ/t = 5 ρ/t, Δ/t = 0.5, and Δ/t = 1−5 ρ/t.

The results reported in Table 1 and Table 2 are for a spherical cavity of radius ρ=0.5 mm and the internal pressure of 10 MPa. The results show that the SCFs are not constant for an incorporated flaw and vary with both the porosity depth and the Poisson ratio, regardless of whether the cylinder geometric ratio is thin or thick. The variation of the SCF in the circumferential direction $K_{t1}$ remains slightly greater than 2, while in the axial direction, the $K_{t2}$ increases slightly with the depth and Poisson ratio $\nu_{m}$; however, it is less than $K_{t3}$. With SCF in the radial direction $K_{t3}$ decreases rapidly with an increasing depth, and it can reach high levels from 43,913 to 36,149 when $\nu_{m}$ is within a range from 0.35 to 0.25. A comparison of the numerical and analytical estimate showed that SCFs fluctuate around an average value ME = 100.4% with a coefficient of variation COV = 0.424%. The comparison showed that a better agreement is provided by Equations (49)–(51) for a spherical cavity subjected to the multiaxial stress field generated by the internal pressure. In addition, this methodology is reliable regardless of the porosity depth, cylinder size, and the material grade.

## 6. Conclusions

Although construction codes may accept a single flaw or a cluster of pores in pressure vessels and pipe welds, these volumetric defects need to meet certain acceptability criteria first of all. These acceptance levels are commonly made only to help engineers make the decision to accept or reject quality of welds, solely based on two parameters: a cavity diameter and a thickness of the tube wall. However, these criteria are not supposed to assess the stress concentration generated by a porosity embedded into the weld of a cylinder. If a flaw in the weld more significant than the quality levels of non-destructive control, rejection is not necessarily automatic and the detailed assessment can be applied. In the present paper, a method allowing determination of exact stress and the corresponding SCFs with the usage of the main equators of the spherical cavity has been applied. The proposed approach allows evaluating the SCFs in terms of three variables: the cylinder geometric ratio, the cavity depth, and the Poisson ratio. Thus, this solution has been validated on two types of a tubes with different geometric ratios, several Poisson ratios and various flaw depths. The developed analytical methodology has provided the results that are in good agreement with the numerical ones.

## Figures and Tables

**Table 1:** Comparison of analytical and finite element analysis (FEA) results of stress concentration factors (SCFs) near the cavity of radius of 0.5 mm and κ = 15.5.

SCF	Δ/t	Proposed Approach (Equations (49)–(51))			FEA Results		
		$\nu_{m}$			$\nu_{m}$		
		0.35	0.3	0.25	0.35	0.3	0.25
$K_{t1}$	5ρ/t	2.190	2.124	2.063	2.181	2.116	2.056
	0.50	2.201	2.134	2.073	2.189	2.124	2.064
	1−5ρ/t	2.212	2.144	2.082	2.217	2.149	2.087
$K_{t2}$	5ρ/t	2.533	2.345	2.174	2.520	2.333	2.162
	0.50	2.568	2.374	2.196	2.550	2.358	2.182
	1−5ρ/t	2.604	2.404	2.220	2.601	2.398	2.213
$K_{t3}$	5ρ/t	43.913	39.855	36.149	43.155	39.165	35.516
	0.50	22.881	20.835	18.967	22.030	20.094	18.310
	1−5ρ/t	15.872	14.496	13.241	15.661	14.307	13.066

**Table 2:** Comparison of analytical and FEA results of SCFs near the porosity of radius of 0.5 mm and κ = 3.1.

SCF	Δ/t	Presented Solution (Equations (49)–(51))			FEA Results		
		$\nu_{m}$			$\nu_{m}$		
		0.35	0.3	0.25	0.35	0.3	0.25
$K_{t1}$	5ρ/t	2.190	2.124	2.063	2.189	2.125	2.066
	0.50	2.297	2.220	2.151	2.304	2.228	2.158
	1−5ρ/t	2.416	2.329	2.249	2.444	2.355	2.227
$K_{t2}$	5ρ/t	2.533	2.345	2.174	2.492	2.305	2.134
	0.50	2.912	2.663	2.427	2.909	2.651	2.414
	1−5ρ/t	3.579	3.201	2.857	3.615	3.232	2.834
$K_{t3}$	5ρ/t	43.913	39.855	36.149	43.519	39.463	35.921
	0.50	6.076	5.638	5.238	5.980	5.549	5.154
	1−5ρ/t	4.115	3.865	3.636	4.095	3.845	3.617

## Data Availability

All data generated or analyzed during this study are included in this published article.

## Nomenclature

$\epsilon_{ij}^{*}\left(X\right)$	Eigenstrain
$\Omega_{m}$ *,* $\Omega_{i}$	Domain occupied by the matrix and the inclusion, respectively
$C_{ijkl}^{m}$ *,* $C_{ijkl}^{i}$	Elastic stiffness of the matrix and the inclusion
$\sigma_{ij}^{0}$ *,* $\epsilon_{ij}^{0}$	Applied stress and the corresponding deformation
$\sigma_{ij}^{d}\left(X\right)$ *,* $\epsilon_{ij}^{d}\left(X\right)$	Total stress and the corresponding deformation
$\sigma_{ij}\left(X\right)$ *,* $\epsilon_{ij}\left(X\right)$	Perturbation stress and the corresponding deformation
$S_{klmn}$	Fourth rank Elshby’s tensor
$\Delta \sigma_{ij}$	Stress jump across the interface
n	Unit vector normal to the interface
${\bar{\sigma }}_{ij}^{0}$ *,* ${\bar{\epsilon }}_{ij}^{0}$	Average applied stress and corresponding deformation
$\sigma_{r}$	Reference stress
P	Applied internal pressure
$r_{i}$ *,* $r_{e}$	Internal and external radius of the tube
$r_{C}$	Cavity position with respect to the cylinder axis
ρ	Porosity radius
$\nu_{m}$ *,* $E_{m}$ *,* $\mu_{m}$	Poisson ratio, Young and shear modulus of the cylinder
$\nu_{i}$ *,* $E_{i}$ *,* $\mu_{i}$	Poisson ratio, Young and shear modulus of the inclusion
$K_{t}$	Stress concentration factor
κ	Geometric ratio of the cylinder
Δ	Cavity depth
d	Distance between two inclusions
t	Cylinder wall thickness

## Appendix A

### Appendix A.1. Expressions of the Coefficients $C_{0}$ to $C_{5}$

Using the Eshelby’s solution, the eigenstrain $\epsilon^{*}$ can be determined in terms of the components of the Eshelby’s tensor for a spherical cavity. However, the dimensionless constants $C_{0}$ to $C_{5}$, which appear in Equations (23)–(25), are found by solving and rearranging Formula (15) into the following form:

$$\epsilon^{*}={\left[S-I\right]}^{-1}\epsilon^{0}$$

where the components of the Eshelby’s tensor are given as follows:

$$S_{1111}=S_{2222}=S_{3333}=\frac{\left(7-5\nu_{m}\right)}{15\left(1-\nu_{m}\right)}\;S_{1122}=S_{2233}=S_{3311}=\frac{\left(5\nu_{m}-1\right)}{15\left(1-\nu_{m}\right)}$$

### Appendix A.2. Components of Normal Constraint $\sigma_{11}$ across a Spherical Porosity

The stresses around the cavity are given in the main equators $x_{11}$, $x_{22}$, and $x_{33}$ by Equation (15). Thus, the expression of the circumferential stress component in the equator $x_{11}$ can be deducted with

$$\sigma_{11}=C_{1111}^{m}\left[-\left(C_{11mn}^{m}\epsilon_{mn}^{*}M_{11}n_{1\;}n_{1}+C_{22mn}^{m}\epsilon_{mn}^{*}M_{12}n_{2\;}n_{1}+C_{33mn}^{m}\epsilon_{mn}^{*}M_{13}n_{3\;}n_{1}\right)+\epsilon_{11}^{*}\right]\;+C_{1122}^{m}\left[-\left(C_{11mn}^{m}\epsilon_{mn}^{*}M_{21}n_{1\;}n_{2}+C_{22mn}^{m}\epsilon_{mn}^{*}M_{22}n_{2\;}n_{2}+C_{33mn}^{m}\epsilon_{mn}^{*}M_{23}n_{3\;}n_{2}\right)+\epsilon_{22}^{*}\right]\;+C_{1133}^{m}\left[-\left(C_{11mn}^{m}\epsilon_{mn}^{*}M_{31}n_{1\;}n_{3}+C_{22mn}^{m}\epsilon_{mn}^{*}M_{32}n_{2\;}n_{3}+C_{33mn}^{m}\epsilon_{mn}^{*}M_{33}n_{3\;}n_{3}\right)+\epsilon_{33}^{*}\right]$$

where $n_{1}=0$, $n_{2}=cos\theta$, $n_{3}=sin\theta$, and the stiffness tensors $C_{ijkl}^{m}$ are provided by the Formula (5). Therefore, the previous equation has the following form:

$$\sigma_{11}=C_{1111}^{m}\epsilon_{11}^{*}+C_{1122}^{m}\left[-\left(C_{22mn}^{m}\epsilon_{mn}^{*}M_{22}\cos^{2}\theta +C_{33mn}^{m}\epsilon_{mn}^{*}M_{23}cos\theta sin\theta \right)+\epsilon_{22}^{*}\right]\;+C_{1133}^{m}\left[-\left(C_{22mn}^{m}\epsilon_{mn}^{*}M_{32}cos\theta sin\theta +C_{33mn}^{m}\epsilon_{mn}^{*}M_{33}\sin^{2}\theta \right)+\epsilon_{33}^{*}\right]$$

where the coefficients $M_{22}$, $M_{23}$, $M_{32}$ and $M_{33}$ can be obtained from Equation (12):

$$M_{22}=\frac{1}{\mu_{m}}\left[1-\frac{n_{2}n_{2}}{2\left(1-\nu_{m}\right)}\right]\;M_{23}=\frac{1}{\mu_{m}}\left[-\frac{n_{2}n_{3}}{2\left(1-\nu_{m}\right)}\right]\;M_{32}=\frac{1}{\mu_{m}}\left[-\frac{n_{3}n_{2}}{2\left(1-\nu_{m}\right)}\right]\;M_{33}=\frac{1}{\mu_{m}}\left[1-\frac{n_{3}n_{3}}{2\left(1-\nu_{m}\right)}\right]$$
